# Generation of expressed sequence tags for discovery of genes responsible for floral traits of *Chrysanthemum morifolium* by next-generation sequencing technology

**DOI:** 10.1186/s12864-017-4061-3

**Published:** 2017-09-04

**Authors:** Katsutomo Sasaki, Nobutaka Mitsuda, Kenji Nashima, Kyutaro Kishimoto, Yuichi Katayose, Hiroyuki Kanamori, Akemi Ohmiya

**Affiliations:** 1 Institute of Vegetable and Floriculture Science, National Agriculture and Food Research Organization (NARO), 2-1 Fujimoto, Tsukuba, Ibaraki 305-0852 Japan; 2 Plant Gene Regulation Research Group, Bioproduction Research Institute, National Institute of Advanced Industrial Science and Technology (AIST), Central 6, 1-1-1 Higashi, Tsukuba, Ibaraki 305-8566 Japan; 3 Institute of Fruit Tree and Tea Science, National Agriculture and Food Research Organization (NARO), 2-1 Fujimoto, Tsukuba, Ibaraki 305-8605 Japan; 4 College of Bioresource Sciences, Nihon University, 1866 Kameino, Fujisawa, Kanagawa 252-0880 Japan; 5 Institute of Crop Science, National Agriculture and Food Research Organization (NARO), 1-2 Owashi, Tsukuba, Ibaraki 305-8634 Japan

**Keywords:** *Chrysanthemum morifolium*, Expressed sequence tag, Next-generation sequencing technology, Transcription factor, Transcriptome

## Abstract

**Background:**

*Chrysanthemum morifolium* is one of the most economically valuable ornamental plants worldwide. Chrysanthemum is an allohexaploid plant with a large genome that is commercially propagated by vegetative reproduction. New cultivars with different floral traits, such as color, morphology, and scent, have been generated mainly by classical cross-breeding and mutation breeding. However, only limited genetic resources and their genome information are available for the generation of new floral traits.

**Results:**

To obtain useful information about molecular bases for floral traits of chrysanthemums, we read expressed sequence tags (ESTs) of chrysanthemums by high-throughput sequencing using the 454 pyrosequencing technology. We constructed normalized cDNA libraries, consisting of full-length, 3′-UTR, and 5′-UTR cDNAs derived from various tissues of chrysanthemums. These libraries produced a total number of 3,772,677 high-quality reads, which were assembled into 213,204 contigs. By comparing the data obtained with those of full genome-sequenced species, we confirmed that our chrysanthemum contig set contained the majority of all expressed genes, which was sufficient for further molecular analysis in chrysanthemums.

**Conclusion:**

We confirmed that our chrysanthemum EST set (contigs) contained a number of contigs that encoded transcription factors and enzymes involved in pigment and aroma compound metabolism that was comparable to that of other species. This information can serve as an informative resource for identifying genes involved in various biological processes in chrysanthemums. Moreover, the findings of our study will contribute to a better understanding of the floral characteristics of chrysanthemums including the myriad cultivars at the molecular level.

**Electronic supplementary material:**

The online version of this article (doi:10.1186/s12864-017-4061-3) contains supplementary material, which is available to authorized users.

## Background

Chrysanthemum (*Chrysanthemum morifolium*) is one of the most commercially important ornamental plants in the world. New chrysanthemum cultivars have been generated mainly by classical cross-breeding and mutation breeding [1–3].

Chrysanthemums are an allohexaploid (2n = 6× = 54), with somatic chromosome numbers varying within the range of 2n = 47–63, and have multiple loci because of a loss or gain of several chromosomes [4, 5]. It is difficult to obtain S1 progeny because, similarly to many other plants that belong to the Asteraceae family, the sporophytic self-incompatibility of chrysanthemums [6] precludes selfing [7, 8]. Therefore, for commercial production, chrysanthemum cultivars have been propagated asexually. In addition, the genome size of chrysanthemum cultivars is enormous (12.381—24.802 Gbp; http://www.etnobiofic.cat/gsad_v2/. Accessed Aug 23, 2017) when compared with those of other model plants such as Arabidopsis (*Arabidopsis thaliana*; 0.125 Gbp) [9] and rice (*Oryza sativa* L.; 0.389 Gbp) [10]. Furthermore, the chimerical structure should be considered in the analysis of ray-petal-color inheritance [11]. Due to these characteristics, obtaining a pure line that is ideal for genome and genetic analyses is difficult, and the progress of the inheritance analysis of ornamental traits is hindered.

Genetic analyses in model plants, such as Arabidopsis and snapdragon (*Antirrhinum majus*), and the reverse genetic analysis in many plant species have revealed that transcription factors (TFs) play important roles in the development of floral organs and floral traits. In the genome of Arabidopsis, 1717 TF loci exist (PlantTFDB v4.0; http://planttfdb.cbi.pku.edu.cn/index.php?sp=Ath, Accessed Aug 23, 2017) [12] which were classified into more than 50 families according to differences in their DNA binding domains [13]. TFs not only determine the floral organ identity (ABC model) [14–16] but also contribute to the development of floral traits, such as morphology, colors, scent, and floral architecture [17–19]. However, the functions of TFs that participate in the identification of floral organs and/or characterization of their ornamental traits in chrysanthemums have been poorly understood, because the information concerning their genomic sequences is scarce, and performing a genetic analysis is difficult.

Ray petal colors of ancestral chrysanthemums are limited to pink, yellow, and white, which derived from anthocyanins and/or carotenoids, and absence of these pigments, respectively [20, 21]. Because flower color is a crucial ornamental trait, much effort has been devoted to the production of novel flower colors [2]. In the modern chrysanthemum cultivars, a wide range of flower colors, including purplish red, orange, red, and dark red, have been developed by increasing the range of pigment content or combinations of these two pigments. Recently, green-flowered cultivars containing chlorophyll in their petals have become popular. However, knowledge of the molecular basis of the control of metabolic pathways of these pigments in the flowers of chrysanthemums is still limited.

Floral scent is another factor that affects the commercial value of chrysanthemum cultivars. Nearly 200 volatile compounds have been identified in the flowers of chrysanthemum cultivars. The main compounds are monoterpenes and oxygenated monoterpenes, such as camphor, α-pinene, chrysanthenone, safranal, myrcene, and eucalyptol [22]. These compounds are not only responsible for the floral scent characteristics of chrysanthemums, but they also provide antioxidant activity in the use of chrysanthemums as herbs [23]. Importantly, an improvement of floral scent properties would be necessary to increase the commercial value of chrysanthemum cultivars. However, the molecular mechanism that regulates such a complex mixture of volatile compounds in chrysanthemums is not elucidated.

Expressed sequence tag (EST) sequencing is important for conducting functional analysis, especially in horticultural plants whose genome information is lacking. Due to the large genome and high genome polyploidy, the complete genome sequence of chrysanthemums is difficult to read. In this study, we performed a large-scale transcriptome analysis using a next-generation sequencer in chrysanthemums and obtained a considerable amount of EST data and more than 200,000 contigs. The contigs contained a series of genes related to pigment and aroma compound metabolism, i.e., TF families which have been previously reported in Arabidopsis. This work will make a significant contribution as a reference for future studies toward a genetic, biochemical, and molecular understanding of the floral traits of chrysanthemums.

## Results and discussion

### EST sequencing and assembly

**Fig. 1:**
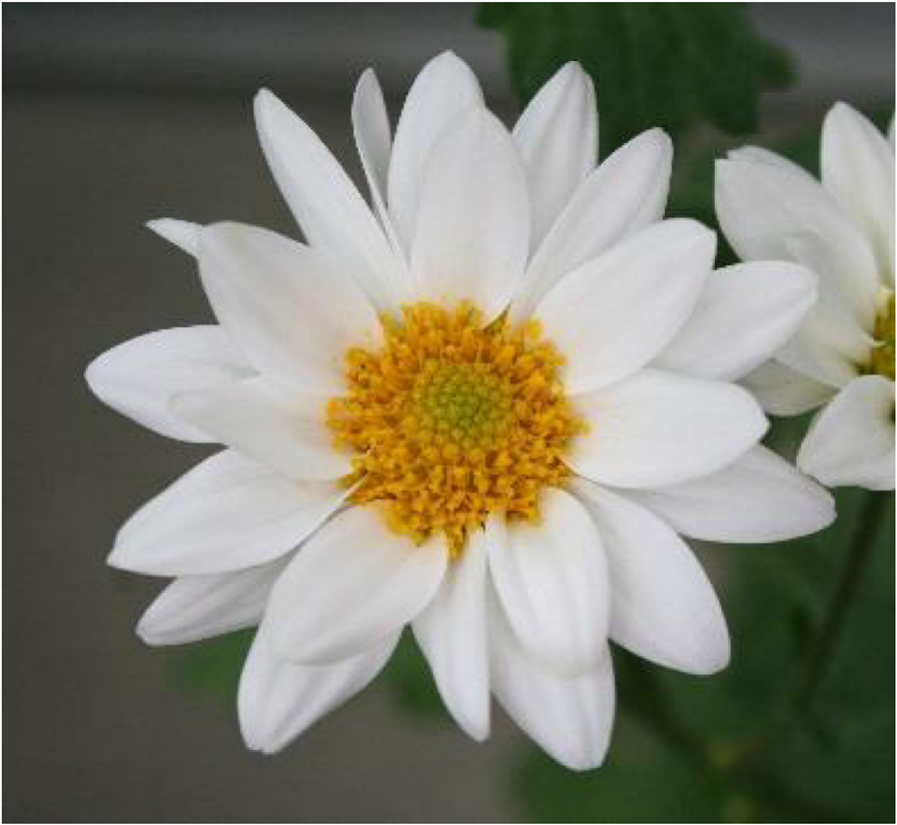
A photograph of a flower of the *Chrysanthemum morifolium* cultivar ‘Sei-Marine’ used in this study

For the EST sequencing, we used the chrysanthemum cultivar ‘Sei-Marine’ (Fig. 1), which has a single-type flower with two types of florets, disk florets, and ray florets. We prepared four independent cDNA libraries to maximize the range of expression transcript diversity (Table 1). These cDNA libraries were produced by RNA mixtures extracted from several vegetative and floral organs at various developmental stages. Because chrysanthemum cultivars are mostly allohexaploid with loss or gain of several chromosomes [4, 5] and their genomes are allopolyploid, the existence of many single-nucleotide polymorphisms (SNPs) was estimated even though the genomic/RNA sequences were derived from only one cultivar. We, therefore, chose to employ the GS FLX 454 system for the EST sequencing, which can read relatively longer DNA sequences, because short-read sequences were supposed to be inadequate for assembling the chrysanthemum ESTs containing abundant SNPs. The 454 sequencing process was performed after the normalization of the following four cDNA libraries. The first cDNA library was prepared by RNAs derived only from floral organs, providing a total number of 1302 k reads. The second, third, and fourth cDNA libraries were prepared by RNAs derived from various aerial organs of the chrysanthemum, including flowers, leaves, stems, and the total numbers of these reads were 761 k, 1001 k, and 708 k, respectively. In particular, the third and fourth cDNA libraries were purified as 3′- and 5′-cDNA libraries to increase the available sequences from the 3′ and 5′ ends of the transcripts. A total of 3773 k reads from these four cDNA libraries were obtained from the 454 sequencing run. The average sequence lengths of these four cDNA libraries were 358 bp, 585 bp, 457 bp, and 470 bp, respectively. Because the cDNA sequences were sufficiently long for the 454 sequencing system, the generated cDNA libraries were supposed to be of good quality. The total numbers of the bases of these cDNA libraries were 466 Mbp, 446 Mbp, 457 Mbp, and 332 Mbp, respectively, and a total of 1702 Mbp bases were sequenced using the 454 sequencing method.

**Table 1:** Summary of chrysanthemum transcript data

Library	1st	2nd	3rd	4th	Total
Organs	Floral organs	Entire organs	Entire organs	Entire organs	
Type of cDNA	full length	full length	3′ purified	5′ purified	-
Total number of reads	1302 k	761 k	1001 k	708 k	3773 k
Average sequence length	358 bp	585 bp	457 bp	470 bp	-
Total bases	466Mbp	446Mbp	457Mbp	332Mbp	1702Mbp

The 3773 k sequences (3,772,677 reads; Table 2) were assembled after trimming and filtering processes. The 3′ and 5′ adaptor sequences, which were used for the construction of these cDNA libraries, were removed from the 3,772,677 sequences. Next, the polyT signals on the 5′-terminal side of the reading sequences were removed. In addition, the polyA signals on the 3′-terminal side of the sequences were also eliminated. The read sequences whose lengths were less than 40 bp after these trimming processes, were excluded from the cDNA assembly, and thus a total of 3,573,676 sequences remained for the assembly. Next, the assembling was performed with MIRA (version 3.4.0) [24] using these 3,573,676 read sequences. Furthermore, the read sequences that matched to a huge larger of other reading sequences and sequences containing short repeats were excluded. Consequently, 3,243,586 reading sequences were used for the assembly, and 213,204 contigs were finally obtained.

**Table 2:** Summary of filtering and assemblies of chrysanthemum transcriptome data

Total Reads	3,772,677
Available reads evaluated after filtering	3,573,676
Invalid reads for assembling	324,723
Singlets that were not used for construction of contigs	5367
Reads used for construction of contigs	3,243,586
Contigs	213,204

### Functional annotation

**Fig. 2:**
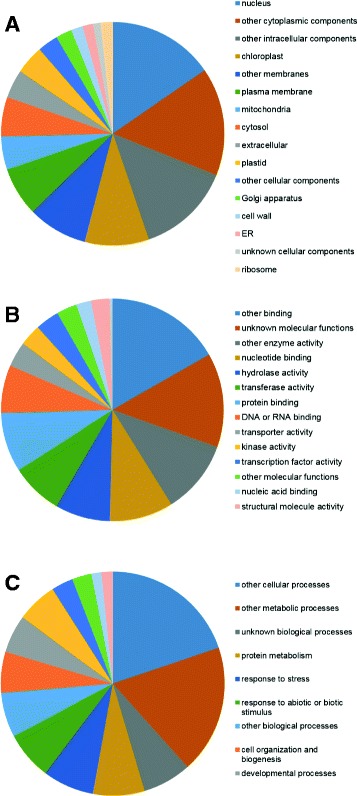
Classification of transcripts into functional categories according to Arabidopsis Gene Ontology. a cellular component; b molecular function; and c biological process

The 213,204 contigs were annotated using the Arabidopsis protein data set (TAIR10; https://www.arabidopsis.org/portals/genAnnotation/gene_structural_annotation/annotation_data.jsp; Accessed Aug 23, 2017) from the Arabidopsis Information Resource (TAIR) (https://www.arabidopsis.org/; Accessed Aug 23, 2017) through the BLASTX program using an E-value cutoff of 1e-5 according to the classification of Gene Ontology (GO). As a result, 61% of the contigs produced top-hits to 16,740 loci of Arabidopsis in total. Because only 15,297 loci were top-hit when all rice genes were employed as BLASTX queries to all Arabidopsis proteins, we considered that our contig set covered the majority of all genes expressed in chrysanthemums. The GO Slim classification of the Arabidopsis gene models was applied to estimate the functional role of each contig. The functional classifications of the contigs that matched Arabidopsis genes within each GO term are shown in Fig. 2. In the molecular function category, “nucleotide binding (9.2%),” “hydrolase activity (8.0%),” “transferase activity (7.6%),” “protein binding (8.6%),” and “DNA or RNA binding (6.9%)” are highly represented terms. In the biological process category, “protein metabolism (7.4%),” “response to stress (7.4%),” “response to abiotic or biotic stimulus (7.0%),” “cell organization and biogenesis (5.9%),” “developmental process (5.5%),” and “transport (5.8%)” are highly represented terms. In the cellular component category, “nucleus (15.4%)” is the most highly represented, and “chloroplast (9.3%)” and “plasma membrane (7.1%)” are highly represented terms. The proportion of genes assigned to GO terms was similar to that of the Arabidopsis gene annotation.

### The concept of gene “cluster”

**Fig. 3:**
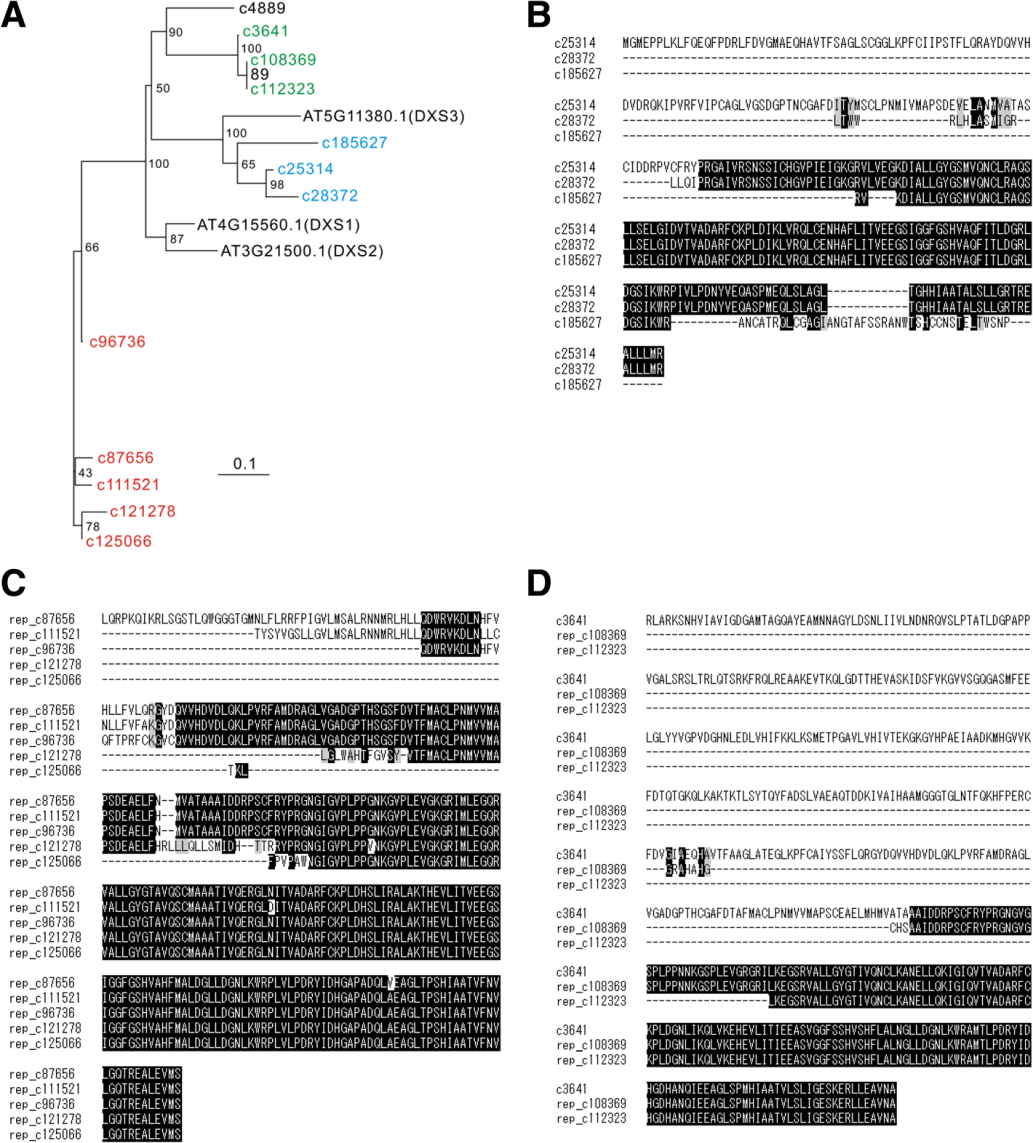
Classification of DXS genes. a Phylogenetic tree of DXS genes of Arabidopsis and chrysanthemum. According to our definition, same-colored contigs are classified into the same cluster. b–d Amino-acid alignment of the contigs in each cluster. Contigs in the same cluster share very high sequence homology

**Fig. 4:**
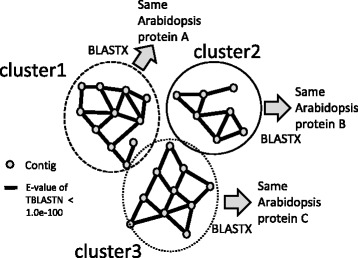
The concept of the clusters. Contigs within the same cluster top-hit to the same Arabidopsis gene with BLAST E-value <1.0e-05 and very high sequence homology (BLAST E-value <1.0e-100) with at least one contig constituting the cluster. In this diagram, 28 contigs are consolidated into three clusters

Because chrysanthemums are allohexaploid, it is generally supposed that three exceedingly similar genes form a type of a “cluster.” In other words, the large number of contigs identified in this study could be consolidated into approximately one-third of the number of gene clusters. For instance, we found 12 putative orthologous contigs of 1-Deoxy-D-xylulose-5-phosphate synthase (DXS) genes in our chrysanthemum contig set, but they were classified into four groups or gene clusters according to their homologies (Fig. 3a). As shown in the alignments, it is natural to speculate that the contigs in each cluster belong to the same gene class derived from different or the same diploid set (Fig. 3a, b, and c). Thus, we attempted to classify ca. 131,500 contigs that had obvious homology to a particular Arabidopsis gene (E-value of BLASTX <1.0e-5), depending on the mutual homology. We defined a gene cluster as a set of contigs which top-hit to the same Arabidopsis gene and had high homology (E-value of TBLASTN <1.0e-100 when the longest open translated reading frame was employed as a query) to at least one of the other contigs in the same cluster (Fig. 4). As a result, we defined 39,221 gene clusters, which were almost one-third (30%) of the input and was comparable to the number of all genes in other plant species (Additional file 1: Table S1). This concept is useful in estimating the number of orthologous gene classes of known genes in chrysanthemums.

### Transcription factors

TFs play important roles not only in the establishment of floral organ identity but also in the characterization of floral traits, such as colors, scent, and morphology. We had previously performed molecular breeding using TF genes to modify these floral traits in floricultural plants, such as torenia, rose, gentian, lisianthus, cyclamen, chrysanthemum, and morning glory [25, 26]. However, until now, the information of chrysanthemum TFs has been poorly characterized. In TFs, the sequences that encode DNA binding domains are relatively conserved among orthologs of other plant species, but the sequence similarity in other regions between different species is generally low. Therefore, cloning the full-length of TFs was difficult by the time of the emergence of the next-generation sequencing technology, and their functional analysis progressed slowly. In this study, we confirmed that all TF families that had been reported in Arabidopsis [13] were also conserved in chrysanthemums (Table 3). Our data sets contain 6368 TF contigs, which are consolidated into 2132 TF clusters (Table 3). For the classification of the TFs in chrysanthemums, we employed the information of Arabidopsis TFs in the PlantTFDB database [12].

**Table 3:** Classification of TF families in chrysanthemum

	TF Family	Contig	Cluster	Arabidopsis
1	AP2	43	18	18
2	ARF	129	57	22
3	ARR-B	22	13	14
4	B3	111	53	66
5	BBR-BPC	42	11	7
6	BES1	38	12	8
7	bHLH	600	179	153
8	bZIP	306	92	74
9	C2H2	352	130	100
10	C3H	216	89	50
11	CAMTA	52	18	6
12	CO-like	80	27	17
13	CPP	14	5	8
14	DBB	68	13	11
15	Dof	112	42	36
16	E2F/DP	17	13	8
17	EIL	28	10	6
18	ERF	644	187	123
19	FAR1	76	57	17
20	G2-like	124	50	42
21	GATA	130	40	30
22	GeBP	49	18	22
23	GRAS	152	71	34
24	GRF	14	9	9
25	HB-other	27	16	7
26	HB-PHD	5	4	2
27	HD-ZIP	223	71	48
28	HRT-like	1	1	2
29	HSF	119	38	24
30	LBD	83	37	43
31	LFY	1	1	1
32	LSD	52	7	3
33	MIKC_MADS	496	62	42
34	M-type_MADS	19	9	66
35	MYB	509	198	144
36	MYB_related	247	86	66
37	NAC	338	118	113
38	NF-X1	12	5	2
39	NF-YA	30	11	10
40	NF-YB	110	19	13
41	NF-YC	52	14	14
42	Nin-like	221	113	14
43	NZZ/SPL	0	0	1
44	RAV	7	4	6
45	S1Fa-like	0	0	3
46	SAP	0	0	1
47	SBP	115	44	17
48	SRS	23	9	11
49	STAT	2	2	2
50	TALE	126	38	21
51	TCP	76	37	24
52	Trihelix	165	48	29
53	VOZ	12	4	2
54	Whirly	9	2	3
55	WOX	20	9	16
56	WRKY	340	124	72
57	YABBY	69	8	6
58	zf-HD	68	22	17
		6996	2375	1726

**Fig. 5:**
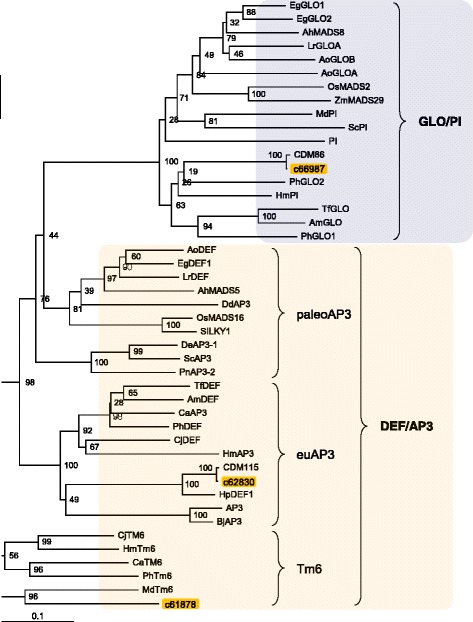
Phylogenetic tree of chrysanthemum class-B MADS-box proteins. Deduced amino-acid sequences of class-B proteins found in the chrysanthemum EST data (Cluster ID of our data) and those in other plant species (Additional file 2: Table S2) were compared and a phylogenetic tree was constructed using the neighbor-joining method. For the phylogenetic analysis, a chrysanthemum contig that was the most homologous to the *Arabidopsis* ortholog at the amino-acid levels was used as a representative of the clusters in this study

To further examine whether TFs specifically expressed in floral organs were included in our chrysanthemum contig sets, we searched the contigs for class-B MADS-box genes, *DEFICIENS* (*DEF*)/*APETALA3* (*AP3*), and *GLOBOSA* (*GLO*)/*PISTILLATA* (*PI*) that play important roles in petal development [27–31]. In chrysanthemums, two class-B genes, *CDM115* (*DEF*/*AP3*) and *CDM86* (*GLO*/*PI*), have been previously registered in the NCBI GenBank, and these genes were also found in our contigs. In addition to *CDM115* and *CDM86*, three class B MADS-box clusters were newly found in our contig sets. Among them, the *TM6* gene [32] of the DEF/AP3 subgroup was identified for the first time in a chrysanthemum. A phylogenetic relationship was examined using class-B MADS-box proteins derived from chrysanthemums and other plant species (Fig. 5). In the phylogenetic analysis, the most homologous contig to Arabidopsis ortholog was used as a representative of the clusters classified in this study. The analysis results suggested that EST data in this study contained all types of class-B subgroups. Chrysanthemum DEF/AP3 was classified into the euAP3 group and possessed C-terminal euAP3 motifs, which are unique for dicots [33]. In petunia, PhDEF and PhTM6 proteins exhibit functional divergence [32]; hence the chrysanthemum TM6 and DEF/AP3 proteins may also have different roles in floral organ development. In chrysanthemums, the function of MADS-box proteins in floral organ-morphogenesis has been poorly understood. Our EST data would contribute to the future research in this area. In addition, our report reveals the causal genes and/or important factors that are involved in formation of floral traits, such as petal shape, floral architecture, and color patterns in various floral types of chrysanthemum cultivars regarding the TF functions.

### Carotenoid metabolism

**Fig. 6:**
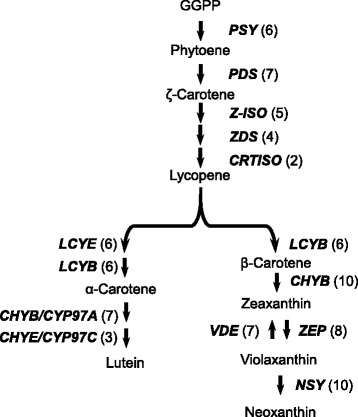
Distribution of chrysanthemum ESTs in the carotenoid biosynthesis pathway. Each enzyme name is followed, in parentheses, by the number of contigs homologous to gene families that encode this enzyme. PSY, phytoene synthase; PDS, phytoene desaturase; Z-ISO, 15-*cis*-ζ-CRTISO; ZDS, ζ-carotene desaturase; CRTISO, carotenoid isomerase; LCYB, lycopene β-cyclase; LCYE, lycopene ε-cyclase; CHYB, β-ring hydroxylase; CHYE, ε-ring hydroxylase; ZEP, zeaxanthin epoxidase; VDE, violaxanthin de-epoxidase; and NSY, neoxanthin synthase

Carotenoids are widely distributed C40 isoprenoid pigments. They play an important role in photosynthesis and provide flowers and fruits with yellow, orange, and red colors. Yellow ray petals of chrysanthemums accumulate substantial amounts of carotenoids, mainly luteins and lutein epoxides [20]. Contigs of all the steps of carotenoid biosynthesis are presented in our chrysanthemum EST data (Fig. 6). Kishimoto et al. [34] reported sequences of full-length cDNAs encoding carotenoid biosynthesis enzymes in chrysanthemums, including phytoene synthase (PSY), phytoene desaturase (PDS), ζ-carotene desaturase (ZDS), carotenoid isomerase (CRTISO), lycopene β-cyclase (LCYB), lycopene ε-cyclase (LCYE), β-ring hydroxylase (CHYB), violaxanthin deepoxidase (VDE), and zeaxanthin epoxidase (ZEP). In addition to contig-encoding these enzymes, we newly identified contigs for 15-*cis*-ζ-CRTISO (Z-ISO), neoxanthin synthase (NSY), and P450-type hydroxylases, CHYB/CYP97A, and ε-ring hydroxylase (CHYE/CYP97C). The EST data contained 2–10 contigs for each enzyme (Fig. 6). Importantly, the sequence comparison revealed that the contigs encoding ZDS, CRTISO, Z-ISO, CHYE/CYP97A, CHYB/CYP97C, VDE, and NSY showed more than 90% identities within each enzyme. Other enzymes, including PSY, PDS, LCYB, LCYE, CHYB, and ZEP had contigs that showed less than 70% identity to the previously reported full-length cDNA sequences [34]. Carotenoid biosynthesis is highly regulated in a tissue-specific manner. The different patterns of carotenogenic gene expression between leaves and petals are partly due to the existence of multiple homologs of rate-limiting enzymes. For example, multiple homologs of *PSY*, *LCYB*, and *CHYB* are present in tomato and are expressed in a tissue-specific manner [35]. It is of great interest to elucidate the tissue specificity of the multiple types of ESTs for the carotenoid biosynthesis enzymes found in the chrysanthemum EST data.

Specific enzymatic cleavage of carotenoids produces various types of apocarotenoids, some of which have important biological functions in the growth and development of plants [36]. In Arabidopsis, enzymes that cleave carotenoids fall into nine clades [37]. Five of these, the 9-*cis* epoxycarotenoid dioxygenases (NCED2, NCED3, NCED5, NCED6, and NCED9), are involved in the biosynthesis of the plant hormone abscisic acid. The remaining four, carotenoid cleavage dioxygenases (CCD1, CCD4, CCD7, and CCD8), have low sequence homologies to the NCEDs, and their enzyme activities and substrate specificities also differ from those of the NCEDs [36]. Orthologs that belong to the NCED3, CCD4, and CCD1 subfamilies have previously been identified in chrysanthemums [38, 39]. In this examination, apart from the ESTs encoding these enzymes, we newly identified contigs for NCED5 and NCED6 in the chrysanthemum EST data. We found only a single short *CCD7* EST, but could not identify *CCD8* EST. CCD7 and CCD8 are involved in the synthesis of the plant hormone strigolactone, which is mainly synthesized in roots [40, 41]. *CCD7* and *CCD8* transcripts were not contained in the EST database, possibly because the EST database was constructed using RNAs obtained from aerial organs.

**Fig. 7:**
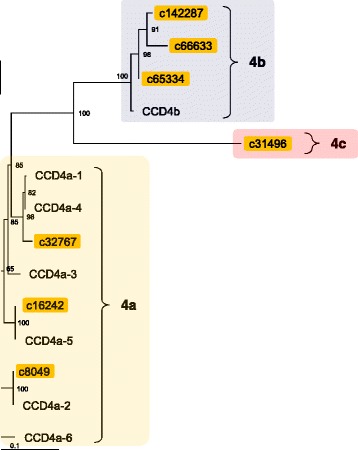
A phylogenetic tree of the chrysanthemum CCD4. Deduced amino-acid sequences of CCD4 found in the chrysanthemum EST database (indicated by EST IDs) and those previously identified in the chrysanthemum cultivar ‘Jimba’ (indicated by GenBank accession numbers in Additional file 4: Table S4) were compared, and a phylogenetic tree was constructed using the neighbor-joining method. 4a, 4b, and 4c indicates CCD4a, CCD4b, and CCD4c subfamilies, respectively

In chrysanthemums, *CCD4a* is expressed specifically in ray petals, and its translated product cleaves carotenoids into colorless compounds, thus resulting in a white petal color [38]. Chrysanthemums have another type of *CCD4* (designated *CCD4b*), which is expressed mainly in leaves and stems, although its function is unknown [38]. We found nine *CCD4a* and seven *CCD4b* contigs. In addition, we newly found seven *CCD4* contigs whose sequences showed low identities (<62%) to both *CCD4a* and *CCD4b* (designated *CCD4c*; Fig. 7). We also performed phylogenetic analyses using amino-acid sequences that were translated by the longest ORF with high homology to a particular Arabidopsis gene (Fig. 7; selection criteria are shown in Methods section). In the chrysanthemum cultivar ‘Jimba’, at least 6 *CCD4a* homologs (*CmCCD4a-1–6*) were identified [39]. In this study, we identified *CCD4a-1*, *−2*, and *−5* homologs in our contig set that was made from cultivar ‘Sei-Marine’ (Fig. 7). In addition, a short *CCD4a-3* contig (DJ0055_rep_c164774) was also detected in the contig set, although it was not used for phylogenetic analysis. These results are consistent with the findings of a previous study that revealed that the number of *CCD4a* homologs expressed in petals differs among chrysanthemum cultivars [42].

### Terpene biosynthesis

**Fig. 8:**
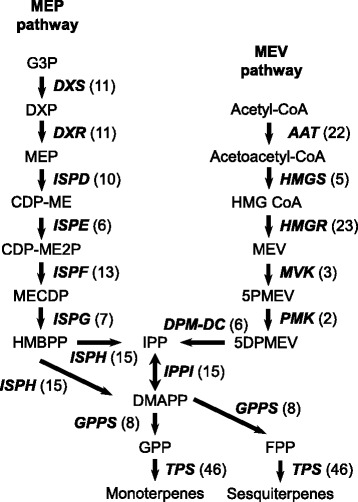
Distribution of chrysanthemum ESTs in the terpenoid biosynthetic pathway. Each enzyme name is followed, in parentheses, by the number of contigs homologous to the gene families that encode this enzyme. MEP, 2-C-Methyl-D-erythritol 4-phosphate; MEV, Mevalonate; G3P, Glyceraldehyde 3-phosphate; DXP, 1-Deoxy*-*D-xylulose 5-phosphate; CDP-ME, 4-(Cytidine 5′-diphospho)-2-C-methyl-D-erythritol; MECDP, 2-C-Methyl-D-erythritol-2,4-cyclodiphosphate; HMBPP, (*E*)-4-Hydroxy-3-methylbut-2-en-1-yl diphosphate; IPP, Isopentenyl diphosphate; DMAPP, Dimethylallyl diphosphate; GPP, Geranyl diphosphate; FPP, Farnesyl diphosphate; HMG-CoA, (*S*)-3-Hydroxy-3-methylglutaryl-CoA; DXS, 1-Deoxy-D-xylulose-5-phosphate synthase; DXR, 1-Deoxy-D-xylulose-5-phosphate reductoisomerase; ISPD, 2-C-Methyl-D-erythritol 4-phosphate cytidylyltransferase; ISPE, 4-(Cytidine 5′-diphospho)-2-C-methyl-D-erythritol kinase; ISPF, 2-C-Methyl-D-erythritol 2,4-cyclodiphosphate synthase; ISPG, 4-Hydroxy-3-methylbut-2-en-1-yl diphosphate synthase; ISPH, 4-hydroxy-3-methylbut-2-enyl diphosphate reductase; IPPI, Isopentenyl diphosphate isomerase; AAT, Acetyl-CoA acetyltransferase; HMGS, HMG-CoA synthase; HMGR, HMG-CoA reductase; MVK, Mevalonate kinase; PMK, Phosphomevalonate kinase; DPM-DC, Diphosphomevalonate decarboxylase; GPPS, GPP synthase or FPP synthase; and TPS, Terpene synthase

The main compounds that characterize the floral scent of chrysanthemums are volatile isoprenoids, such as monoterpenes (C10) and oxygenated monoterpenes [22]. We established that sesquiterpenes (C15) are also present as minor compounds. These low-molecular-weight terpenes are enzymatically synthesized from the 5-carbon precursors isopentenyl diphosphate and its isomer, dimethyl diphosphate, both of which are derived from the methylerythritol phosphate (MEP) pathway or the mevalonate (MEV) pathway (Fig. 8) [43]. We investigated genes that encoded enzymes that belonged to the terpene-biosynthesis pathway in our chrysanthemum EST database and found that all the genes were included in the database (Fig. 8). The EST data contained 2–46 contigs for each enzyme. In higher plants, the putative numbers of terpene synthase (TPS) genes per genome range from approximately 20 to 150 [44]. In the chrysanthemum EST, we identified 46 *TPS* contigs, which were consolidated into 30 clusters. Thus, these numbers of clusters were fell within the range (20 to 150) (Fig. 8) [44]. Many chrysanthemum contigs that encoded MEP and MEV pathway enzymes were highly homologous to those of *Artemisia annua* that belonged to Asteraceae.

**Fig. 9:**
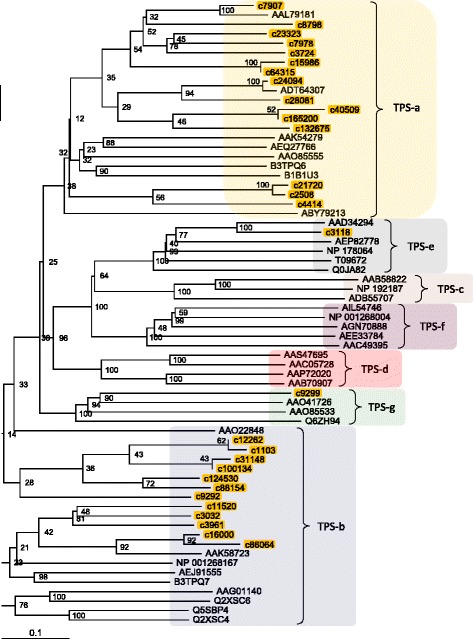
A phylogenetic tree of terpene synthase (TPS). Deduced amino-acid sequences of chrysanthemum (Cluster ID of our data) and those of similar protein family members (Genbank accession number in Additional file 5: Table S5) previously identified in higher plants were compared, and a phylogenetic tree was constructed using the neighbor-joining method. For the phylogenetic analysis, the chrysanthemum contig that was the most homologous to the Arabidopsis ortholog at the amino-acid level was used as a representative of the clusters in this study

The TPS family is responsible for the synthesis of various monoterpene compounds. We identified a total of 46 highly homologous contigs that encoded TPS that were combined into 30 clusters. Figure 9 represents the phylogenetic tree based on amino-acid sequence comparisons of TPS between chrysanthemums and other plant species. In the phylogenetic analysis, the most homologous contig to the Arabidopsis ortholog was used as a representative of the clusters classified in this study. Of the 30 clusters defined, 29 clusters were used in the phylogenetic analysis because one cluster (contig) had only short amino-acid sequences (the amino-acid length was <20% of the alignment coverage). Currently, seven major TPS subfamilies, designated TPS-a through TPS-g, are recognized in plants [44–48]. Most of the chrysanthemum TPS appeared to fall into the same clades as TPS-a or -b subfamilies, except for one EST that was classified into the TPS-g subfamily. This result was consistent with the previously reported data that TPS-a, −b, and -g subgroups are specific to angiosperms.

TPSs have an ability to synthesize multiple terpenes from a single substrate. The chemical diversity of the terpenes produced in chrysanthemum flowers may be mainly attributed to such unique enzyme activity of TPS. Future studies should focus on the identification and functional characterization of the *TPS* genes involved in the floral scent formation in chrysanthemums.

## Conclusion

In this study, we performed an EST sequencing analysis in a chrysanthemum and obtained 213,204 assembled contigs. These contigs were further consolidated into 39,221 clusters, which were comparable to the number of all genes in other plant species. In addition, the contigs in this study contained a comparable number of TFs and genes involved in petal colors and aroma compound biosynthesis. These results suggest that our EST data set is a valuable reference for the molecular analysis of chrysanthemums. Chrysanthemums have a large and allohexaploid genome, which hinders genomic sequencing. However, the recent progress in sequencing technology may facilitate the genomic sequencing of chrysanthemums in the near future. However, genomic structures and/or sequences are different among chrysanthemum cultivars due to their self-incompatibility, and each cultivar is not a pure line. Therefore, continuous efforts by using EST and/or genomic sequencing analyses of multiple chrysanthemum cultivars are necessary for further understanding of the chrysanthemum genome. We believe that our EST analysis that employs the concept of gene clusters could help to elucidate not only the specifics of chrysanthemum genes but also those of other higher polyploid plants.

## Methods

### Plant materials and RNA extraction

A *Chrysanthemum morifolium* cultivar ‘Sei-Marine’ (provided by Inochio Seikoen Co., Ltd.; http://www.seikoen-kiku.co.jp/) was used in this study. The chrysanthemum was grown in a greenhouse under natural daylight conditions. We used the following plant organs as materials for sequencing analysis: mature corolla of ray floret and immature corolla of ray floret (including pistils) obtained from 5 to 8 mm flower buds, mature corolla of disk floret and immature corolla of disk floret (including pistils and androecium) obtained from 5 to 8-mm flower buds, shoot apices, flower buds under 4 mm, stems, leaves, phyllary, blossom end, pistils of ray florets and disk florets, and androecia of disk florets. These materials were prepared from several independent chrysanthemum plant lines and/or flowers. Total RNAs from these organs were extracted using TRIzol (Thermo Fisher Scientific; https://www.thermofisher.com/jp/ja/home.html). The total RNAs were further purified by an RNeasy mini-kit (QIAGEN; https://www.qiagen.com/jp/). RNA concentration was estimated with an ND-1000 spectrophotometer (NanoDrop) (Thermo Scientific; Wilmington, DE, USA).

### Preparations of cDNA libraries for 454 sequencing

For 454 sequencing, a normalized cDNA library was prepared. Total RNAs isolated from each chrysanthemum organ were combined in an attempt to maximize the diversity of the transcriptional units and were used as a material for cDNA synthesis. We used the following four independent cDNA libraries (Table 1).

For the first cDNA library, the cDNA was prepared using total RNAs derived from floral organs. The cDNA synthesis and normalization were performed by the following procedure. The first strand cDNA synthesis of the first library was synthesized with the following oligonucleotide: 5′-CAAGCAGAAGACGGCATACGACTGGAGTTTTTTTTTTTTTTTTVN-3′. Next, the diol group of the synthesized cDNA/RNA hybrid was oxidized, followed by biotinylation. After digestion with RNase I, the cDNA/RNA hybrid was purified using the cap-trapping method, and full-length single-strand cDNAs were obtained. A 5′ adaptor (Additional file 6: Table S6) was attached to the cap-trapping single-strand cDNAs, and second-strand cDNAs were synthesized using the following oligonucleotide: 5′-AATGATACGGCGCTGGAGGACAGGTTCAGAGTTC-3′. Further, the prepared double-strand cDNA was heat-denatured; the re-hybridized double-strand cDNAs indicated highly accumulated transcripts in the library that were digested with double-strand-specific DNA nuclease. The single-strand cDNAs, which were obtained by the above manipulation, were collected as normalized cDNAs. Next, second-strand cDNAs were synthesized using the normalized single-strand cDNAs.

The cDNAs for the second, third, and fourth cDNA libraries were synthesized using total RNAs derived from the aerial part of the chrysanthemum with a Mint-2 cDNA synthesis kit (evrogen; http://evrogen.com/) according to the manufacturer’s instructions. The first strand cDNAs were synthesized utilizing the following oligonucleotide: 5′-attctagaggccgaggcggccgacatgTTTTTTTTTTTTTTTV-3′. After the second-strand cDNA synthesis, the cDNA libraries were amplified using a primer set consisting of 3′ PCR primer 2 (5′-ATTCTAGAGGCCGAGGcggccgac-3′) and 5′ PCR primer (5′-aagcagtggtatcaacgcagagt-3′) under the following conditions: 95 °C for 2 min, followed by 18 cycles of 95 °C for 15 s, 66 °C for 20 min, and 72 °C for 6 min. The amplified cDNA libraries were normalized by the TRIMMERDIRECT cDNA Normalization Kit (evrogen) according to the manufacturer’s guidelines. The normalized cDNA libraries were further amplified using the primer sets respective to each type of the cDNA libraries. We prepared three types of normalized cDNA libraries derived from the aerial part of chrysanthemum: (1) full-length cDNA libraries (second), (2) 3′ cDNA library (third), and (3) 5′ cDNA library (fourth). The full-length second-cDNA library was amplified using the 3′ PCR primer 2 and the 5′ PCR primer under the following conditions: 95 °C for 2 min, followed by 16 cycles of 95 °C for 15 s, 66 °C for 20 min, and 72 °C for 6 min. The third- and fourth cDNA libraries were also amplified using the 3′ PCR primer 2 and the 5′ PCR primer under the following conditions: 95 °C for 2 min, followed by 17 cycles of 95 °C for 15 s, 66 °C for 20 min, and 72 °C for 6 min. A biotin-labeled 3′ PCR primer 2 was used for the amplification of the 3′ cDNA library. A biotin-labeled 5′ PCR primer was used for the amplification of the 5′ cDNA library. These biotin-labeled cDNA libraries were purified through Dynabeads M-280 Streptavidin (Thermo Fisher Scientific) according to the manufacturer’s instructions. The biotin-labeled cDNA libraries were purified and recovered as single-strand cDNAs, and double-strand DNAs were synthesized by KOD-Plus-Ver.2 (TOYOBO; http://www.toyobo-global.com/). The isolated double-strand DNAs were fragmented to an approximate length of 800 bp and subjected to sequencing analysis by the LE220 Focused-ultrasonicator (Covaris; http://covarisinc.com/).

### Sequencing and data analysis

Sequencing was performed by the 454 FLX titanium system (first library; Table 1) and the GS FLX+ system (second, third, and fourth libraries; Table 1) (Roche; https://roche-biochem.jp/index.html) according to the manufacturer’s instructions. The 454 sequences were trimmed of adaptor and low-quality sequences. The sequences were then assembled and annotated by BLASTN searches of the NCBI database.

### Functional annotation

To annotate the contig sequences, each contig was searched against the Arabidopsis protein database (TAIR10) from The Arabidopsis Information Resources (TAIR; http://www.arabidopsis.org) using the BLASTX program with an E-value cut-off of 1e-5. Annotation of the best-hit Arabidopsis protein was used as an annotation of each contig. Functional classification of the contigs was performed based on the Gene Ontology (GO) terms from the Arabidopsis GO Slim classification in TAIR.

### Phylogenetic analyses and selection of chrysanthemum orthologs

For the phylogenetic analyses, protein sequences were aligned using MAFFT (http://mafft.cbrc.jp/alignment/software/; Accessed Aug 23, 2017). After these sequences had been aligned, they were used for phylogenetic analysis by the neighbor-joining method in Clustal W. Statistical significance was tested by bootstrap analysis for 1000 replicates. Amino-acid sequences of other plant species were obtained from NCBI (http://www.ncbi.nlm.nih.gov/nuccore; Accessed Aug 23, 2017, see Additional files 1, 3, 4, and 5 for accession numbers: Table S1, S2, S3, and S5). The phylogenetic analyses were performed using the chrysanthemum amino-acid sequences, which were translated using the longest ORF with high homology to a particular Arabidopsis gene (threshold E-value of BLASTX <1.0e-5, threshold score > 150, and minimum alignment coverage of 20% of the query sequences).

Chrysanthemum ortholog genes in Figs. 6 and 8 were also selected using a threshold E-value of BLASTX <1.0e-5, a threshold score > 150, and minimum alignment coverage of 20% of the query sequences.

### Accession numbers

Raw sequencing data of chrysanthemum with the 454 sequencing were submitted to the DDBJ Sequence Read Archive (DRA; http://trace.ddbj.nig.ac.jp/dra/index.html; Accessed Aug 23, 2017) with the accession numbers (DRR088291-DRR088294). Assembled transcripts derived from the raw sequencing data were further submitted to the Mass Submission System of DDBJ (MSS; http://www.ddbj.nig.ac.jp/sub/mss_flow-j.html; Accessed Aug 23, 2017) with accession numbers IABW01000001–IABW01213204 (213,204 entries).

## Additional files

Additional file 1: Table S1. Annotations of contigs and clusters in this study. (XLSX 18507 kb)

Additional file 2: Table S2. Gene accessions used in a phylogenetic tree of class-B proteins. (XLSX 26 kb)

Additional file 3: Table S3. Gene accessions used in the phylogenetic tree of CCD4 proteins. (XLSX 12 kb)

Additional file 4: Table S4. Codes for searching the homologous contigs in chrysanthemum. (XLSX 14 kb)

Additional file 5: Table S5. Gene accessions used in the phylogenetic tree of TPS proteins. (XLSX 28 kb)

Additional file 6: Table S6. Sequences of an adaptor. (XLSX 8 kb)

## Abbreviations

AAT: Acetyl-CoA acetyltransferase
AP3: APETALA3
CCD: Carotenoid cleavage dioxygenases
CDP-ME: 4-(Cytidine 5′-diphospho)-2-C-methyl-D-erythritol
CHYB: β-ring hydroxylase
CHYE: ε-ring hydroxylase
CRTISO: Carotenoid isomerase
DEF: DEFICIENS
DMAPP: Dimethylallyl diphosphate
DPM-DC: Diphosphomevalonate decarboxylase
DXP: 1-Deoxy*-*D-xylulose 5-phosphate
DXR: 1-Deoxy-D-xylulose-5-phosphate reductoisomerase
DXS: 1-Deoxy-D-xylulose-5-phosphate synthase
EST: Expressed sequence tag
FPP: Farnesyl diphosphate
G3P: Glyceraldehyde 3-phosphate
GLO: GLOBOSA
GO: Gene ontology
GPP: Geranyl diphosphate
GPPS: GPP synthase
HMBPP: (*E*)-4-Hydroxy-3-methylbut-2-en-1-yl diphosphate
HMG-CoA: (*S*)-3-Hydroxy-3-methylglutaryl-CoA
HMGR: HMG-CoA reductase
HMGS: HMG-CoA synthase
IPP: Isopentenyl diphosphate
IPPI: Isopentenyl diphosphate isomerase
ISPD: 2-C-Methyl-D-erythritol 4-phosphate cytidylyltransferase
ISPE: 4-(Cytidine 5′-diphospho)-2-C-methyl-D-erythritol kinase
ISPF: 2-C-Methyl-D-erythritol 2,4-cyclodiphosphate synthase
ISPG: 4-Hydroxy-3-methylbut-2-en-1-yl diphosphate synthase
ISPH: 4-hydroxy-3-methylbut-2-enyl diphosphate reductase
LCYB: Lycopene β-cyclase
LCYE: Lycopene ε-cyclase
MECDP: 2-C-Methyl-D-erythritol-2,4-cyclodiphosphate
MEP: Methylerythritol phosphate
MEV: Mevalonate
MVK: Mevalonate kinase
NCED: 9-*cis* epoxycarotenoid dioxygenases
NSY: Neoxanthin synthase
PDS: Phytoene desaturase
PI: PISTILLATA
PMK: Phosphomevalonate kinase
PSY: Phytoene synthase
SNP: Single-nucleotide polymorphism
TF: Transcription factor
TPS: Terpene synthase
VDE: Violaxanthin de-epoxidase
ZDS: ζ-carotene desaturase
ZEP: Zeaxanthin epoxidase
Z-ISO: 15-*cis*-ζ-CRTISO
