# Correction to: Generation of expressed sequence tags for discovery of genes responsible for floral traits of *Chrysanthemum morifolium* by next-generation sequencing technology

**DOI:** 10.1186/s12864-017-4206-4

**Published:** 2017-12-07

**Authors:** Katsutomo Sasaki, Nobutaka Mitsuda, Kenji Nashima, Kyutaro Kishimoto, Yuichi Katayose, Hiroyuki Kanamori, Akemi Ohmiya

**Affiliations:** 1 Institute of Vegetable and Floriculture Science, National Agriculture and Food Research Organization (NARO), 2-1 Fujimoto, Tsukuba, Ibaraki, 305-0852 Japan; 2 Plant Gene Regulation Research Group, Bioproduction Research Institute, National Institute of Advanced Industrial Science and Technology (AIST), Central 6, 1-1-1 Higashi, Tsukuba, Ibaraki, 305-8566 Japan; 3 Institute of Fruit Tree and Tea Science, National Agriculture and Food Research Organization (NARO), 2-1 Fujimoto, Tsukuba, Ibaraki, 305-8605 Japan; 4 College of Bioresource Sciences, Nihon University, 1866 Kameino, Fujisawa, Kanagawa 252-0880 Japan; 5 Institute of Crop Science, National Agriculture and Food Research Organization (NARO), 1-2 Owashi, Tsukuba, Ibaraki, 305-8634 Japan

## Correction

After publication of the original article [1] the authors noted that the following errors had occurred:

- Figures 5, 7 and 9 are incomplete in the original article. The updated, full figures are included in this Correction.
- In the section Transcription factors, the last two sentences at the end of the first paragraph contain some incorrect information.
  The following information has been corrected and is visible in the sentence below:
  - “all” TF families has been corrected to “almost all”
  - 6368 TF contigs has been corrected to 6996 TF contigs
  - 2132 TF clusters has been revised to 2375 TF clusters

**Fig. 5:**
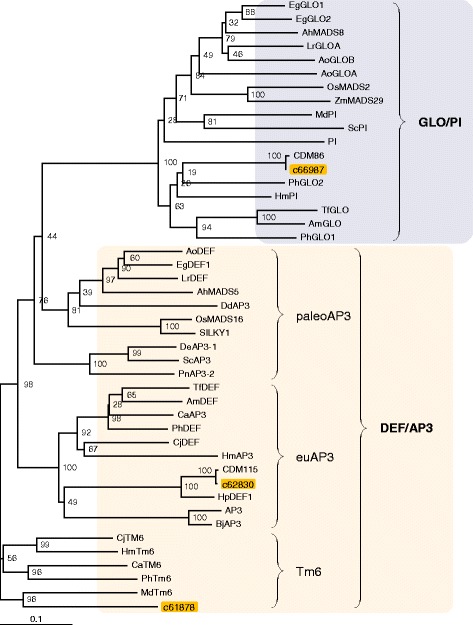
Phylogenetic tree of chrysanthemum class-B MADS-box proteins. Deduced amino-acid sequences of class-B proteins found in the chrysanthemum EST data (Cluster ID of our data) and those in other plant species (Additional file 2: Table S2) were compared and a phylogenetic tree was constructed using the neighbor-joining method. For the phylogenetic analysis, a chrysanthemum contig that was the most homologous to the Arabidopsis ortholog at the amino-acid levels was used as a representative of the clusters in this study

**Fig. 7:**
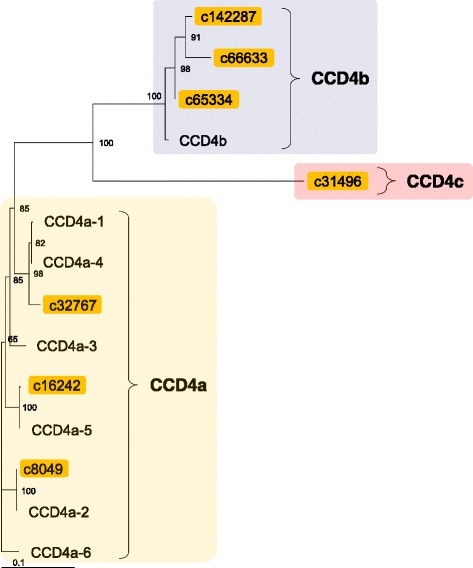
A phylogenetic tree of the chrysanthemum CCD4. Deduced amino-acid sequences of CCD4 found in the chrysanthemum EST database (indicated by EST IDs) and those previously identified in the chrysanthemum cultivar ‘Jimba’ (indicated by GenBank accession numbers in Additional file 4: Table S4) were compared, and a phylogenetic tree was constructed using the neighbor-joining method. 4a, 4b, and 4c indicates CCD4a, CCD4b, and CCD4c subfamilies, respectively

**Fig. 9:**
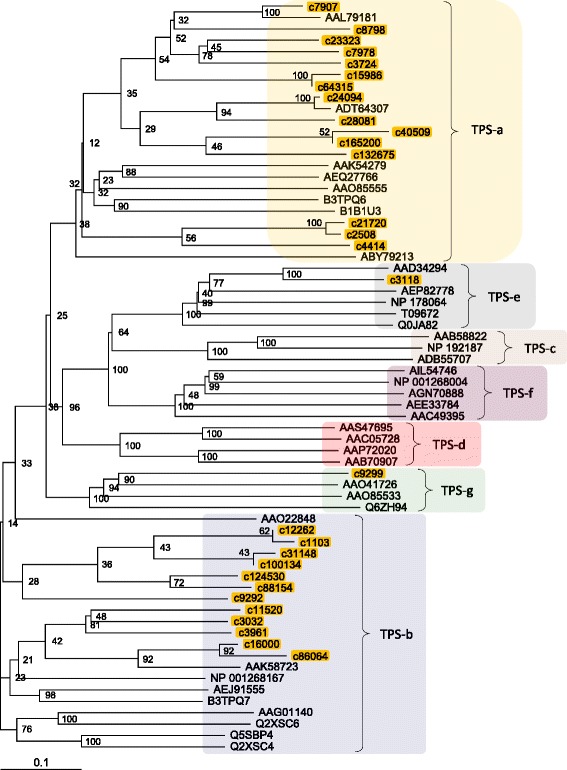
A phylogenetic tree of terpene synthase (TPS). Deduced amino-acid sequences of chrysanthemum (Cluster ID of our data) and those of similar protein family members (Genbank accession number in Additional file 5: Table S5) previously identified in higher plants were compared, and a phylogenetic tree was constructed using the neighbor-joining method. For the phylogenetic analysis, the chrysanthemum contig that was the most homologous to the Arabidopsis ortholog at the amino-acid level was used as a representative of the clusters in this study

In this study, we confirmed that **almost all** TF families that had been reported in Arabidopsis [13] were also conserved in chrysanthemums (Table 3). Our data sets contain **6996** TF contigs, which are consolidated into **2375** TF clusters (Table 3). For the classification of the TFs in chrysanthemums, we employed the information of Arabidopsis TFs in the PlantTFDB database [12].

- The sentence “We identified a total of 46 highly homologouscontigs that encoded TPS that were combined into 30 clusters” should have been removed from the section **“Terpene biosynthesis”.**
  The original article has also been updated.
